# Patient-Reported Dysphagia in Adults with Eosinophilic Esophagitis: Translation and Validation of the Swedish Eosinophilic Esophagitis Activity Index

**DOI:** 10.1007/s00455-021-10277-5

**Published:** 2021-03-08

**Authors:** Sofie Albinsson, Lisa Tuomi, Christine Wennerås, Helen Larsson

**Affiliations:** 1 Department of Infectious Diseases, Institute of Biomedicine, Sahlgrenska Academy, University of Gothenburg, Guldhedsgatan 10A, 413 46 Gothenburg, Sweden; 2 Department of Otorhinolaryngology, Head and Neck Surgery, Institute of Clinical Sciences, Sahlgrenska Academy, University of Gothenburg, Gröna Stråket 9, 413 45 Gothenburg, Sweden; 3 Department of Otorhinolaryngology, Sahlgrenska University Hospital, Gröna Stråket 5, 413 45 Gothenburg, Region Västra Götaland Sweden; 4 Department of Clinical Microbiology, Sahlgrenska University Hospital, Guldhedsgatan 10, 413 46 Gothenburg, Region Västra Götaland Sweden; 5 Department of Otorhinolaryngology, Head and Neck Surgery, NU Hospital Group, Lärketorpsvägen, 461 73 Trollhättan, Region Västra Götaland Sweden

**Keywords:** EEsAI, Patient-reported outcome measurement, Validation, Eosinophilic esophagitis, Deglutition, Deglutition disorders

## Abstract

The lack of a Swedish patient-reported outcome instrument for eosinophilic esophagitis (EoE) has limited the assessment of the disease. The aims of the study were to translate and validate the Eosinophilic Esophagitis Activity Index (EEsAI) to Swedish and to assess the symptom severity of patients with EoE compared to a nondysphagia control group. The EEsAI was translated and adapted to a Swedish cultural context (S-EEsAI) based on international guidelines. The S-EEsAI was validated using adult Swedish patients with EoE (*n* = 97) and an age- and sex-matched nondysphagia control group (*n* = 97). All participants completed the S-EEsAI, the European Organization for Research and Treatment of Cancer Quality of Life Questionnaire-Oesophageal Module 18 (EORTC QLQ-OES18), and supplementary questions regarding feasibility and demographics. Reliability and validity of the S-EEsAI were evaluated by Cronbach’s alpha and Spearman correlation coefficients between the domains of the S-EEsAI and the EORTC QLQ-OES18. A test–retest analysis of 29 patients was evaluated through intraclass correlation coefficients. The S-EEsAI had sufficient reliability with Cronbach’s alpha values of 0.83 and 0.85 for the “visual dysphagia question” and the “avoidance, modification and slow eating score” domains, respectively. The test–retest reliability was sufficient, with good to excellent intraclass correlation coefficients (0.60–0.89). The S-EEsAI domains showed moderate correlation to 6/10 EORTC QLQ-OES18 domains, indicating adequate validity. The patient S-EEsAI results differed significantly from those of the nondysphagia controls (*p* < 0.001). The S-EEsAI appears to be a valid and reliable instrument for monitoring adult patients with EoE in Sweden.

## Background

Eosinophilic esophagitis (EoE) is an immune-mediated esophageal disease [1, 2] characterized histologically by eosinophil-predominant inflammation of the esophageal mucosa and clinically by symptoms related to esophageal dysfunction [3]. The incidence (7/100,000) and prevalence (43/100,000) of EoE in adults in Europe, North America and Australia have increased over time [4], and in Sweden the prevalence among adults has been estimated to be as high as 0.4% in a population-based study [5]. In adults, the most common symptoms are esophageal dysphagia and food impaction [6–8], and many of the affected individuals alter their eating habits by eating very slowly, cutting food into tiny pieces, and drinking copious amounts of water to facilitate the swallowing process [9]. The disease is chronic and requires treatment in the form of dietary restrictions, proton pump inhibitors and/or topical corticosteroids to alleviate symptoms and to prevent fibrotic evolution of this inflammatory condition [3].

Until now, there have been no validated Swedish patient-reported outcome (PRO) instruments for adult patients with EoE, which has limited proper assessment of the disease and made it difficult to compare Swedish research in this area with published international studies. Instead, instruments focused on the dysphagia of esophageal cancer, e.g., the European Organisation for Research and Treatment of Cancer Quality of Life Questionnaire–Oesophageal Module 18 (EORTC QLQ-OES18) [10], or the evaluation of general dysphagia, e.g., the Watson Dysphagia Scale, have been used for the evaluation of symptom severity [11–14]. The EORTC QLQ-OES18 and the Watson Dysphagia Scale have been useful in measuring symptom severity in adult patients with EoE before and after treatment with topical corticosteroids [11, 12]. Additionally, certain items in the EORTC QLQ-OES18 and the Watson Dysphagia Scale were discriminatory in multivariate modeling aimed at separating patients with EoE who were histologic responders to treatment from nonresponders [14].

The Eosinophilic Esophagitis Activity Index (EEsAI) is a PRO instrument specifically developed for the assessment of symptom severity in adult patients with EoE [15]. The instrument is focused on the symptomatic aspects of EoE, including the adaptive behavior that hallmarks the disease. The items that compose this PRO instrument are mainly focused on different food consistencies, including how difficult they are to swallow, and the modifications and adaptions that are required during consumption of the same food consistencies. The EEsAI was originally developed in Switzerland via international collaborations and is currently available in English, French and German [15].

This study aimed to translate, adapt and validate the EEsAI for use in Swedish-speaking adults. A secondary aim was to assess swallowing-related symptom severity in a group of Swedish patients with EoE in comparison with a nondysphagia control group by using the S-EEsAI and the EORTC QLQ-OES18.

## Methods

### Respondents

Adult patients with a confirmed diagnosis of EoE according to the latest diagnostic criteria (symptoms of esophageal dysfunction and ≥ 15 eosinophils/high power field) [3] were recruited at the Ear, Nose and Throat Department of the NU Hospital Group, Trollhättan, Sweden. The desired size of the patient cohort was 95 based on the requirement for validation presented by Fayers and Machin [16], according to which five to ten respondents should be included per item in the evaluated instrument. Patients under 18 years of age, patients with other diseases known to cause dysphagia, and non-Swedish speakers were excluded from the study. For each patient, an age- and sex-matched control individual with no history of esophageal diseases was also recruited; these were recruited among colleagues and acquaintances of the authors. The study participants were recruited by initial contact via telephone, which was confirmed by written informed consent. The study was approved by the Regional Ethical Review Board of Gothenburg, Sweden.

### Study Instruments

The S-EEsAI, combined with the EORTC QLQ-OES18 for evaluation of construct validity, and 20 supplementary questions regarding demographic properties and evaluation of the instruments were filled out by all study participants. The participants could choose whether to answer the instruments manually or electronically. If the instruments had not been filled out within two weeks, a reminder was sent out by e-mail or ordinary mail. Twenty-nine randomly chosen patients from the original patient cohort were asked to complete the set of instruments again two weeks later to provide data for a test–retest evaluation [16].

#### EEsAI

The EEsAI was developed specifically for the assessment of symptom severity in adult patients with EoE and focuses on dysphagia and behavioral adaption during a seven-day interval [15]. It consists of 10 items divided into five domains: visual dysphagia question (VDQ); avoidance, modification and slow eating score (AMS); frequency of trouble swallowing (Frequency); duration of trouble swallowing (Duration); and pain when swallowing (Pain). In the VDQ domain, the patient evaluates the difficulty of swallowing (graded 0–3) eight different food consistencies: solid meat, soft foods, boiled rice, ground meat, white bread, porridge, raw fibrous foods (e.g., apple), and French fries. The AMS domain consists of four items that concern the same eight food consistencies as in the VQD domain but includes yes/no questions regarding avoidance, consumption, modification, and slow eating. The last three domains, i.e., Frequency, Duration and Pain, are single-item domains. Frequency is answered on a 0–3 scale ranging from never to every day; Duration evaluates the typical length of an episode of trouble swallowing using a 0–4 scale ranging from no trouble to time needed to swallow > 5 min; and Pain is a “yes or no” item. All domain scores are subsequently calculated into a final PRO score according to the EEsAI scoring manual [15]. The PRO score ranges from 0 to 100, with a high score indicating severe symptoms. The instrument includes two additional items regarding jaw injuries and explanation for any deviances in the VDQ domain, but they are not included in the PRO score. A summary of the items and the domain structure is provided in Table 1. The validation of the original EEsAI showed that the PRO score adequately reflects the patients’ own assessment of disease severity [15].

**Table 1:** Brief overview of the contents of the items included in the S-EEsAI and their corresponding domains

Item	Question
1	Do you have difficulties chewing?
Visual dysphagia question	
2	Today, how difficult are the eight different foods^a^ to swallow?
3	Explain any deviances in item 2
Avoidance, modification and slow eating score	
4	In the past 7 days, have you altogether avoided these foods^a^ because of your disease?
5	In the past 7 days, have you eaten these foods?^a^
6	In the past 7 days, have you modified these foods?^a^
7	In the past 7 days, have you eaten these foods^a^ slower compared to other people?
Frequency of trouble swallowing	
8	In the past 7 days, how often have you had trouble swallowing?
Duration of trouble swallowing	
9	In the past 7 days, how long did an episode of trouble swallowing last?
Pain when swallowing	
10	In the past 7 days, has it been painful to swallow?

*S-EEsAI* Swedish Eosinophilic Esophagitis Activity Index

^a^The respondents evaluated the difficulty of eating the following eight food consistencies: solid meat, soft foods, boiled rice, ground meat, white bread, porridge, raw fibrous foods, and French fries

The EEsAI was forward–backward translated from English to Swedish according to international and World Health Organization (WHO) recommendations [17, 18]. Two native Swedish speakers who were competent in the English language and had knowledge regarding medical care and terminology provided independent Swedish translations of the English EEsAI. The translations were combined into a consensus version by a three-person expert panel with extensive knowledge of the field and of translation and adaption of questionnaires. The instrument contained adaptions to the Swedish language and culture. The cultural adaptions included the exchange of “grits” to “porridge/oatmeal”, summarization of “dry rice (grains don’t stick) or stocky Asian rice” to “boiled rice”, and the adaption of soft foods to be exemplified by “pudding, omelet and mash”, instead of “pudding, jelly, and apple sauce”. Finally, the consensus version was retranslated into English in a backward translation by an independent bilingual, English native speaker, unfamiliar with the instrument. A pilot study including 10 patients was performed where the patients filled out the instruments, and within a week after the submission, they answered a semistructured interview over the phone. The interview contained general and open questions regarding the instrument and how it was perceived by the patients, such as questions regarding the phrasing, understandability and relevance of the items. Based on the interviews, the patients sought clearer instructions regarding the levels of difficulties of swallowing. The S-EEsAI was accordingly modified with extended exemplification on the definition of mild difficulties in the VDQ domain to include “slow passage of food when swallowing”.

#### EORTC QLQ-OES18

The EORTC QLQ-OES18 is an 18-item instrument designed to assess quality of life of patients with esophageal cancer during a seven-day interval with focus on swallowing difficulties [10]. The items are divided into 10 domains, of which six are single-item domains. The included domains are taste, speech, cough, dry mouth, choking, swallowing saliva, pain, reflux, dysphagia, and eating. All items are answered on a 1–4 scale ranging from “not at all” to “very much”. The answers are transformed into a score ranging from 0 to 100, and each domain is evaluated separately, where a high score represents a high level of discomfort. Validation of the EORTC QLQ-OES18 showed that the instrument is well-accepted and that it demonstrates good psychometric and clinical validity [10].

In this study, the EORTC QLQ-OES18 was included to evaluate the construct validity of the S-EEsAI by analyzing the Spearman correlations between the domains of the two instruments [16].

#### Supplementary Items

Study-specific questions regarding participant demographics and the feasibility of the S-EEsAI were included in the study. The feasibility was evaluated based on the time needed to fill out the instruments and assessed whether any items were difficult to understand or missing, whether any item caused unease, or whether the respondents needed help to fill out the instruments. Questions regarding the occurrence of swallowing difficulties and recent food impaction were also provided to the nondysphagia control group. A question regarding self-assessment of disease severity, graded 0–10 ranging from “no difficulties” (0) to “worst possible difficulties” (10), was provided to the patients with EoE.

### Statistical Analyses

All statistical analyses were performed using IBM SPSS Statistics software, version 25 (IBM, Armonk, NY, USA). *p* < 0.05 was considered statistically significant. Demographic and clinical characteristics of patients and nondysphagia controls were presented using descriptive statistics as percentages for categorical values and as the means for continuous variables.

#### Reliability and Reproducibility

Reliability was evaluated by internal consistency using Cronbach’s alpha coefficient and by evaluation of the Pearson correlation coefficient between each item and its respective domain. Cronbach’s alpha values > 0.7 were considered to indicate satisfactory internal consistency [16]. Pearson correlation coefficients of ≤ 0.39 were considered weak, 0.4–0.59 moderate, and ≥ 0.6 strong correlations [19].

Reliability was further evaluated through test–retest analysis to obtain intraclass correlation coefficients (ICCs) using the two-way mixed-effects model and absolute agreement. An ICC value > 0.75 was considered to reflect excellent reliability, and ICC values of 0.4–0.75 were considered to indicate good reliability [20]. The calculations were performed using data from 29 individuals who completed the instruments twice during a two-week interval; no substantial change in symptom burden of the patients was expected to occur during this time span.

#### Validity

Construct validity includes convergent and discriminant validity and refers to whether the instrument measures the intended construct, i.e., the underlying concept of the outcome [16]. Construct validity was analyzed by determining the Spearman correlations between the domains of the S-EEsAI and the EORTC QLQ-OES18. Prior to the analyses, we hypothesized that the VDQ domain would be moderately correlated with the dysphagia, eating, choking, cough and pain domains of the EORTC QLQ-OES18. We also hypothesized that the AMS domain would be moderately correlated with the dysphagia, eating, choking, cough and pain domains. The Frequency domain was hypothesized to be moderately correlated with the dysphagia, eating, choking, cough, swallowing saliva and pain domains. Finally, the Duration domain was believed to be moderately correlated with the eating and pain domains and the Pain domain with the EORTC QLQ-OES18 pain domain. Spearman correlation coefficients > 0.7 were considered to be strong, 0.3–0.7 were considered to be moderate, and < 0.3 were considered to indicate weak correlations [21]. Comparison of the self-assessment score of disease severity and the PRO score was also performed using Spearman correlations.

Ceiling effects, i.e., the fraction of respondents that provided the maximum score, and conversely, floor effects, the fraction of respondents who provided the minimum score for an item, were also evaluated [22].

The S-EEsAI scores from patients and the nondysphagia control group were compared to evaluate the ability of the instrument to separate esophagus-healthy individuals from patients with EoE. Scores from the EORTC QLQ-OES18 were also compared between patients and the nondysphagia control group. The two-tailed Mann–Whitney *U* test was used for the analyses.

## Results

### Study Participants

**Fig. 1:**
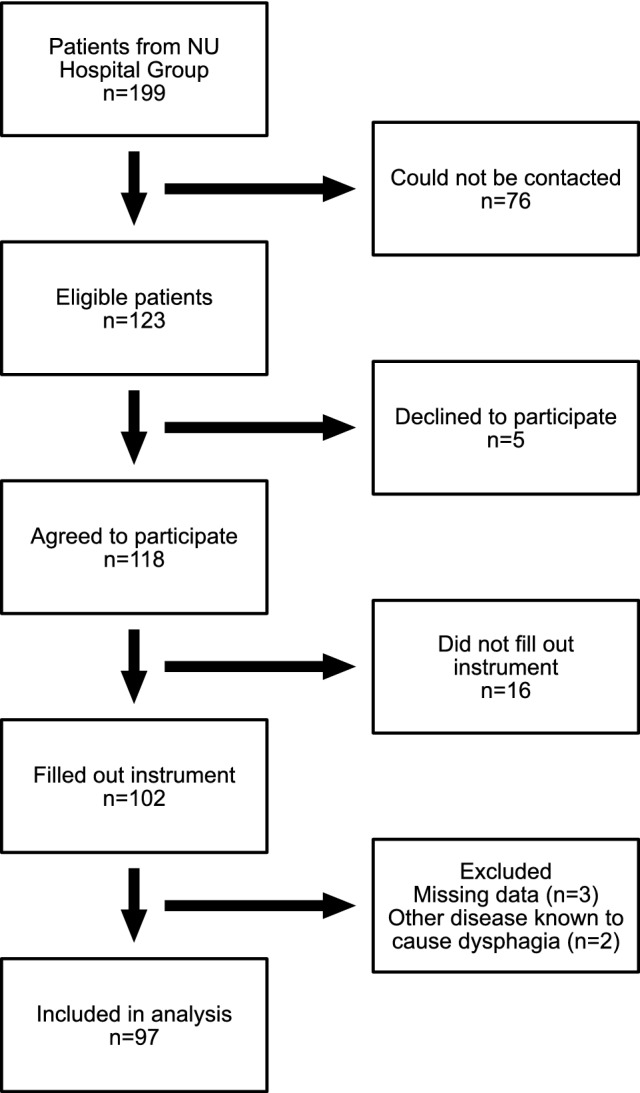
Flow chart of the study patient recruitment process. The chart demonstrates the step-by-step enrollment process whereby 97 out of 199 available adult patients with eosinophilic esophagitis participated in the validation study. The chart was created using Affinity Designer

The current cohort of adult patients with EoE at the NU Hospital Group consisted of 199 patients, 97 of whom were included in the final study (Fig. 1). An age- and sex-matched nondysphagia control group was also recruited (*n* = 97). Sociodemographic and clinical data regarding the participants are presented in Table 2. At the time of the study, 47 (49%) of the patients with EoE were undergoing treatment for EoE. The most common types of treatment were topical corticosteroids and/or oral proton pump inhibitors.

**Table 2:** Sociodemographic and clinical characteristics of the study participants

	Eosinophilic esophagitis patients *n* (%)	Nondysphagia control group *n* (%)	*p* value
Age (years)	52 (16)^a^	52 (16)^a^	NA
Body Mass Index (BMI)	27 (3.7)^a^	26 (3.6)^a^	0.11^b^
Sex			NA
Male	74 (76%)	74 (76%)	
Female	23 (24%)	23 (24%)	
Marital status			0.72^c^
Single	14 (14%)	18 (19%)	
Married/cohabitating	77 (79%)	74 (76%)	
Partnership but living apart	6 (6.2%)	5 (5.2%)	
Working status			0.40^c^
Fulltime	70 (72%)	80 (83%)	
Part time	8 (8.2%)	8 (8.2%)	
Retired	15 (16%)	8 (8.2%)	
Unemployed/job searching/other	4 (4.1%)	1 (1.0%)	
Smoking			0.18^d^
Never smoked	64 (66%)	55 (57%)	
Have quit smoking	31 (32%)	38 (40%)	
Current smoker	2 (2.1%)	3 (3.1%)	
Time to complete survey			< 0.001^c^
< 10 min	26 (27%)	65 (67%)	
10–20 min	60 (63%)	31 (32%)	
> 20 min	10 (10%)	1 (1.0%)	

*NA* not applicable

^a^Mean (standard deviation)

^b^Mann–Whitney *U* test

^c^Chi-2 test

^d^Mantel–Haenszel test

### Feasibility

The instruments were completed either manually (patients *n* = 30; nondysphagia controls *n* = 9) or electronically (patients *n* = 67; nondysphagia controls *n* = 88). Although the majority of the patients completed all three instruments within 10–20 min, the time to complete the survey was shorter for the nondysphagia control group, which was the only statistically significant difference between the groups (Table 2). Four patients reported that the VDQ domain of the S-EEsAI was difficult to answer for the following reasons: no current swallowing difficulties due to medical treatment, difficulty remembering food not recently ingested, or a wish for a larger span of scoring alternatives than the four provided levels. Additionally, in the paper version of the S-EEsAI, the workflow of the AMS domain was sometimes interpreted differently by the study participants due to ambiguity of the layout and when data were missing, the responses had to be omitted from the analyses.

### Reliability and Reproducibility

Internal consistency was determined to evaluate whether the items of each domain of the S-EEsAI measured the underlying concepts of dysphagia and behavioral adaption. Both the VDQ and AMS domains demonstrated satisfactory reliability regarding internal consistency with Cronbach’s alpha values of 0.83–0.85 (Table 3). Furthermore, the test–retest procedure revealed excellent/good test–retest reliability with ICC values of 0.60–0.89 (Table 3).

**Table 3:** Reliability estimates of the S-EEsAI based on 97 Swedish patients with eosinophilic esophagitis

Domain	Cronbach's alpha	ICC
Visual dysphagia question	0.83	0.89
Avoidance, modification and slow eating score	0.85	0.60
Patient-reported outcome score	NA	0.89

*ICC* intraclass correlation coefficient; *NA* not applicable; *S-EEsAI* Swedish Eosinophilic Esophagitis Activity Index

Pearson correlations between the S-EEsAI items, including all investigated food consistencies of the item, and their assigned domains were investigated to evaluate whether the items had been placed in the proper domain (Table 4). For the VDQ domain, 7/8 food consistencies displayed moderate to strong correlations (*r* = 0.46–0.65). For the AMS domain, moderate to strong correlations were noted for 20/24 food consistencies among the three items (*r* = 0.41–0.68). It was also seen that a total of five food consistencies in items 2, 6 and 7 were more strongly correlated to other domains compared to their own domains, which might be an indication of inappropriate item placement, namely, “2-porridge”, “6-boiled rice”, “6-white bread”, “7-ground meat”, and “7-French fries” (Table 4). No correlations were calculated between the AMS domain score and item 5, since this item only concerned the types of foods that have been consumed during the preceding 7 days, which formed the basis for the food consistencies that were to be considered in the subsequent items.

**Table 4:** Pearson correlation between S-EEsAI domains and items, including the consistencies of each item

Item	VDQ	AMS	Frequency	Duration	Pain	*n* (%)
1	NA	NA	NA	NA	NA	96 (99)
2-Solid meat	0.65**	0.40**	0.58**	0.58**	0.11	97 (100)
2-Soft foods	0.48**	0.26*	0.32*	0.27*	0.099	97 (100)
2-Boiled rice	0.49**	0.28*	0.30*	0.31*	0.16	97 (100)
2-Ground meat	0.50**	0.17	0.38**	0.40**	0.13	97 (100)
2-White bread	0.49**	0.23*	0.23*	0.23*	0.11	97 (100)
2-Porridge	0.17	0.14	0.21*	0.11	0.086	97 (100)
2-Raw fibrous food	0.46**	0.31*	0.36**	0.38**	0.092	97 (100)
2-French fries	0.63**	0.28*	0.44**	0.33*	0.15	97 (100)
4-Solid meat	0.27*	0.68**	0.13	0.19	− 0.0022	97 (100)
4-Soft foods	− 0.12	0.41**	− 0.089	− 0.17	0.10	97 (100)
4-Boiled rice	0.43**	0.60**	0.21*	0.23*	− 0.030	97 (100)
4-Ground meat	0.19	0.56**	0.082	0.17	0.10	97 (100)
4-White bread	0.25*	0.46**	0.12	0.18	0.12	97 (100)
4-Porridge	0.083	0.28*	0.10	− 0.037	0.017	97 (100)
4-Raw fibrous food	0.31*	0.67**	0.15	0.15	0.069	97 (100)
4-French fries	0.25*	0.49**	0.18	0.15	0.12	97 (100)
6-Solid meat	0.41**	0.58**	0.52**	0.28*	0.25*	75 (77)
6-Soft foods	0.16	0.38**	0.21	0.20	0.15	77 (79)
6-Boiled rice	0.013	0.26	0.15	0.13	0.37*	49 (51)
6-Ground meat	0.28*	0.41**	0.16	0.10	0.15	67 (69)
6-White bread	0.19	0.30*	0.28*	0.34*	0.025	72 (74)
6-Porridge	0.46**	0.60**	0.33*	0.34*	0.15	50 (52)
6-Raw fibrous food	0.34*	0.53**	0.27*	0.19	0.10	61 (63)
6-French fries	0.28	0.55**	0.30	0.13	0.0051	40 (41)
7-Solid meat	0.47**	0.60**	0.49**	0.42**	0.22	75 (77)
7-Soft foods	0.47**	0.64**	0.43**	0.34*	0.33*	77 (79)
7-Boiled rice	0.47**	0.60**	0.39*	0.38*	0.16	49 (51)
7-Ground meat	0.53**	0.49**	0.53**	0.57**	0.32*	67 (69)
7-White bread	0.56**	0.61**	0.57**	0.42**	0.41**	71 (73)
7-Porridge	0.44*	0.54**	0.38*	0.24	0.54**	51 (53)
7-Raw fibrous food	0.48**	0.62**	0.41*	0.27*	0.28*	60 (62)
7-French fries	0.52**	0.49*	0.49*	0.30	0.24	40 (41)
8-Frequency	NA^a^	NA^a^	NA^a^	NA^a^	NA^a^	97 (100)
9-Duration	NA^a^	NA^a^	NA^a^	NA^a^	NA^a^	97 (100)
10-Pain	NA^a^	NA^a^	NA^a^	NA^a^	NA^a^	97 (100)
VDQ	NA	0.58**	0.68**	0.59**	0.27*	97 (100)
AMS	0.58**	NA	0.46**	0.40**	0.23*	97 (100)
PRO	0.81**	0.69**	0.86**	0.71**	0.58**	97 (100)

The number and percentage of patients with eosinophilic esophagitis who answered each item are listed in the final column

*AMS* avoidance, modification and slow eating score; *Duration* duration of trouble swallowing; *Frequency* frequency of trouble swallowing; *NA* not applicable; *Pain* pain when swallowing; *PRO* patient-reported outcome score; *S-EEsAI* Swedish Eosinophilic Esophagitis Activity Index; *VDQ* visual dysphagia question

Items 2, 4, 6, and 7 are presented for each of the eight food consistencies included in the item

^a^Single-item domain, correlation between item and corresponding domain is always 1, and comparison with other domains was therefore excluded

Weak correlation < 0.39, moderate correlation 0.40–0.59, strong correlation > 0.60 [19]

^*^p < 0.05, **p < 0.001

Not all items were answered by all individuals (missing data), and the number of answers per item is listed in Table 4.

### Validity

Construct validity was determined by calculating the Spearman correlation between the S-EEsAI domains and the domains of the EORTC QLQ-OES18 (Table 5). The VQD, AMS, and Frequency domains and the PRO score of the S-EEsAI correlated moderately with the dysphagia domain of the EORTC QLQ-OES18 (*r* = 0.33–0.42). Moderate correlations were also found between all S-EEsAI domains and the EORTC QLQ-OES18 choking domain (*r* = 0.31–0.55). All but the Pain domain of S-EEsAI were moderately correlated (*r* = 0.62–0.68) with the EORTC QLQ-OES18 eating domain. Both instruments contain a domain that measures pain, and these domains were moderately correlated (*r* = 0.41).

**Table 5:** Spearman correlation between S-EEsAI domains and EORTC QLQ-OES18 domains based on results from patients with eosinophilic esophagitis

	S-EEsAI					
	VDQ	AMS	Frequency	Duration	Pain	PRO
EORTC QLQ-OES18						
Dysphagia	0.42**	0.36**	0.33*	0.28*	0.068	0.41**
Swallowing saliva	0.28*	0.34*	0.27*	0.14	0.087	0.30*
Choking	0.39**	0.31*	0.55**	0.40**	0.40**	0.51**
Eating	0.68**	0.62**	0.65**	0.62**	0.28*	0.67**
Dry mouth	0.11	0.091	0.038	0.046	0.16	0.076
Taste	0.072	0.077	− 0.0045	− 0.12	− 0.0093	− 0.012
Cough	0.20*	0.29*	0.031	− 0.027	0.22*	0.18
Speech	0.30*	0.15	0.30*	0.28*	0.12	0.28*
Reflux	0.19	0.056	0.15	0.087	− 0.020	0.15
Pain	0.23*	0.19	0.37**	0.23*	0.41**	0.37**

*AMS* avoidance, modification and slow eating score; *Duration* duration of trouble swallowing; *EORTC QLQ-OES18* European Organization for Research and Treatment of Cancer Quality of Life Questionnaire-Oesophageal Module 18; *Frequency* frequency of trouble swallowing; *Pain* pain when swallowing; *PRO* patient-reported outcome score; *S-EEsAI* Swedish Eosinophilic Esophagitis Activity Index; *VDQ* visual dysphagia question

Weak correlation < 0.3, moderate correlation 0.3–0.7, strong correlation > 0.7 [21]

^*^p < 0.05, **p < 0.001

Items that were answered on a scale of more than two options (i.e., not yes/no questions) were checked for skewed distribution of scores regarding both too large fraction of minimal scores (floor effect) and too large fraction of maximal scores (ceiling effect), as shown in Table 6. Floor values predominated, i.e., respondents reporting no swallowing difficulties, which was seen for all items (29–78%). The item that demonstrated the highest floor effect was item 2-soft foods.

**Table 6:** S-EEsAI score distributions of the eosinophilic esophagitis patient cohort. Items based on “yes or no” questions are not included

Item/domain	Grading options	Range (Min–Max)	Median (IQR)	Floor *n* (%)	Ceiling *n* (%)
2-Solid meat	0–3	0–3	1.0 (0.0–2.0)	29 (30)	10 (10)
2-Soft foods	0–3	0–2	0.0 (0.0–0.0)	76 (78)	0.0 (0.0)
2-Boiled rice	0–3	0–3	1.0 (0.0–2.0)	39 (40)	5.0 (5.2)
2-Ground meat	0–3	0–2	1.0 (0.0–2.0)	47 (49)	0.0 (0.0)
2-White bread	0–3	0–2	0.0 (0.0–1.0)	58 (60)	0.0 (0.0)
2-Porridge	0–3	0–2	0.0 (0.0–1.0)	69 (71)	0.0 (0.0)
2-Raw fibrous food	0–3	0–3	1.0 (0.0–2.0)	42 (43)	6.0 (6.2)
2-French fries	0–3	0–3	1.0 (0.0–1.5)	46 (47)	1.0 (1.0)
Frequency	0–3^a^	0–3	1 (0.0–1)	28 (29)	10 (10)
Duration	0–4^a^	0–4	1.0 (0.0–3.0)	29 (30)	6.0 (6.2)
VDQ	0–10^a^	0–7.1	1.7 (0.42–3.8)	NA	NA
AMS	0–10^a^	0–10	1.5 (0.40–3.3)	NA	NA
PRO	0–100	0–83	30 (12–47)	NA	NA

*AMS* avoidance, modification and slow eating score; *Duration* duration of trouble swallowing; *Frequency* frequency of trouble swallowing; *IQR* interquartile range; *NA* not applicable; *PRO* patient-reported outcome score; *S-EEsAI* Swedish Eosinophilic Esophagitis Activity Index; *VDQ* visual dysphagia question

^a^Domain scores before the values are converted for the calculation of the final PRO score

Finally, a significant moderate correlation (*r* = 0.68, *p* < 0.001; Spearman correlation) was observed between the self-assessment score of disease severity (supplementary question for the patient group) and PRO score. The mean value of the self-assessment score for the patients with eosinophilic esophagitis was 2.7 with a standard deviation of 1.8.

### Comparison of Patients with EoE and the Nondysphagia Control Group

To compare the symptom severity of patients with EoE to the nondysphagia control group, summarizing statistics for each group were determined for both the S-EEsAI and the EORTC QLQ-OES18; the final PRO scores and domain scores are summarized in Table 7. The S-EEsAI domain scores and PRO scores were found to be significantly different between the patient and nondysphagia control groups, where the nondysphagia control group received lower scores. Likewise, the EORTC QLQ-OES18 domain scores were significantly different between the groups except for the taste domain (Table 7).

**Table 7:** Comparison of calculated and weighted final scores between patients with eosinophilic esophagitis and nondysphagia controls for S-EEsAI and EORTC QLQ-OES18

	Patients mean (SD)	Nondysphagia control mean (SD)	Fold change	*p* value^a^
S-EEsAI				
VDQ	12 (7.3)	0.87 (3.1)	14	< 0.001
AMS	2.1 (5.6)	0.26 (2.5)	8.1	< 0.001
Frequency	14 (11)	1.1 (3.9)	13	< 0.001
Duration	0.37 (1.5)	0.0 (0.0)	–	0.013
Pain	3.2 (6.2)	0.15 (1.5)	22	< 0.001
PRO	32 (22)	2.4 (7.3)	13	< 0.001
EORTC QLQ-OES18				
Dysphagia	6.7 (11)	6.6 (23)	1.0	0.001
Swallowing saliva	14 (27)	2.4 (13)	5.8	< 0.001
Choking	20 (28)	4.8 (12)	4.2	< 0.001
Eating	18 (21)	1.8 (5.1)	10	< 0.001
Dry mouth	16 (26)	6.5 (14)	2.5	0.0049
Taste	1.7 (7.3)	1.0 (7.6)	1.7	0.26
Cough	13 (22)	6.8 (16)	1.9	0.020
Speech	4.4 (13)	1.0 (5.7)	4.4	0.026
Reflux	18 (22)	7.2 (15)	2.5	< 0.001
Pain	12 (14)	3.2 (6.4)	3.8	< 0.001

*AMS* avoidance, modification and slow eating score; *Duration* duration of trouble swallowing; *EORTC QLQ-OES18* European Organization for Research and Treatment of Cancer Quality of Life Questionnaire-Oesophageal Module 18; *Frequency* frequency of trouble swallowing; *Pain* pain when swallowing; *PRO* patient-reported outcome score; *SD* standard deviation; *S-EEsAI* Swedish Eosinophilic Esophagitis Activity Index; *VDQ* visual dysphagia question

S-EEsAI calculated scores: VDQ: 0–23; AMS: 0–25; Frequency: 0–31; Duration: 0–6; Pain: 0–15; PRO: 0–100 [15]. Each of the EORTC QLQ-OES18 domains are scored from 0 to 100 [10]

^a^Mann–Whitney *U* test

The prevalence of dysphagia in the nondysphagia control group was investigated using supplementary questions. Among the nondysphagia controls, 12% (*n* = 12) reported occasional swallowing difficulties, and 5.2% (*n* = 5) had experienced a recent food impaction. These study participants were offered a voluntary medical checkup regarding their swallowing difficulties.

## Discussion

A validated PRO instrument for adult patients with EoE in Swedish is needed to improve the monitoring and assessment of symptom severity of adults afflicted by this disease. The EEsAI has been deemed one of the best suited instruments for the evaluation of PROs for EoE [23]. Therefore, this study aimed to translate and analyze the psychometric properties of the S-EEsAI.

The internal consistency of the data collected by the instrument as tested by Cronbach’s alpha proved to be satisfactory for both the VDQ and AMS domains. The ICC values calculated from the test–retest were sufficient for PRO and VDQ scores and somewhat lower for the AMS score. The lower ICC value for AMS is most likely affected by item 5, which concerns whether an individual has eaten each of the eight food consistencies or not. The type of food eaten during one week might naturally differ from the food consumed two weeks thereafter, thus affecting the AMS score. Overall, the ICC values showed that the instrument is reliable and reveals similar results when tested repeatedly.

The analysis of correlation between items and domains revealed that some food consistencies of items 2, 4, and 6 correlated more strongly to another domain than its own. The common denominator of these consistencies is softer, untextured foods. Such foods are generally not as difficult for patients with EoE to swallow compared to bulky and textured food [24], which reduces the need to modify and/or avoid them. Additionally, the severity of perceived dysphagia has been found to increase with increasing texture and solidity of the food [15]. This is supported by our results based on the newly validated S-EEsAI, which showed that meat generated the highest scores, followed by raw fibrous foods including apples. This can result in high domain scores even though the scores for soft-textured foods are low.

Floor effects were demonstrated for all items and most commonly for “2-soft foods” and “2-porridge”. The presence of floor effects could indicate that the item should be revised or omitted from the instrument. However, not all patients experience difficulties with the same foods, and even though solid meats are usually the most troublesome to swallow for patients with EoE, some patients also have difficulties with softer foods. This is why it is also useful to investigate these types of foods. The abundance of floor effects might also be due to the current status of the disease, since the state of EoE fluctuates over time [25], or because of ongoing treatment [26]. Accordingly, one patient commented that S-EEsAI questions were difficult to answer because of the current lack of symptoms, as the instrument focuses on symptoms experienced during the preceding 7 days.

Convergent validity was investigated by Spearman correlations between the S-EEsAI and the EORTC QLQ-OES18. The S-EEsAI domains correlated, as hypothesized, moderately to the dysphagia, choking and eating domain, but there were fewer moderate or strong correlations than anticipated. The results demonstrate that, even though some of the domains measure similar constructs, the instruments are not interchangeable. The PRO score was also compared to a supplementary self-assessment score of disease severity and resulted in a moderately strong correlation, which indicates that the PRO score of the S-EEsAI is a good reflection of current disease state in patients with EoE.

Scores generated from an esophagus-healthy population were calculated to provide a basis for comparison of both the S-EEsAI and EORTC QLQ-OES18. This comparison between domain scores and final PRO scores of patients and the nondysphagia control group showed that the instruments are specific and can discriminate patients with EoE from nondysphagia controls.

The feasibility of the S-EEsAI was satisfactory since the time to complete all of the instruments was < 20 min for almost 90% of the patients and the amount of missing data was low. However, the ambiguity of the layout in the AMS domain of the paper version reduces the feasibility and user-friendliness of the instrument. One possibility for increased user-friendliness of the EEsAI presented by Schoepfer et al. [15] was the use of an electronic version. Indeed, the electronic version of the S-EEsAI proved to be more user-friendly since the participants did not need to interpret which items to respond to and because the items in question were adjusted automatically based on the previous response. Additionally, the electronic version did not allow any missing data and facilitated the computation of the PRO score. A larger proportion of the nondysphagia control group used the electronic version, which is probably why the nondysphagia control group answered the instruments more quickly than the patients. The difficulty interpreting which items to respond to in the paper version is a limitation, which is why the electronic version should be recommended for clinical use.

The study may be limited by the inclusion of a nondysphagia control group from colleagues and acquaintances of the authors. It is possible that the results would have been different if the nondysphagia control group had consisted of a random sample of individuals with varying socioeconomic backgrounds similar to the patient group. However, the patient and nondysphagia control group were well-matched regarding age and sex, which are the sociodemographic factors that differ between the EoE patient group and the general population [1]. There are, however, no reports indicating that socioeconomic backgrounds differ between patients with EoE and the general population.

Implementation of the S-EEsAI in Swedish health care will hopefully prove useful for the assessment of symptom severity in newly diagnosed patients with EoE, as well as for long term follow-up of patients with EoE. The instrument could help to determine whether treatment is working properly or not, if dosage adjustments are needed and if treatment should be resumed in patients off treatment.

In conclusion, the results from the validation of the S-EEsAI suggest that the instrument has sufficient reliability and validity and can be used to assess symptom severity in adult patients with EoE in Sweden.
